# Survival probability of stochastic processes beyond persistence exponents

**DOI:** 10.1038/s41467-019-10841-6

**Published:** 2019-07-05

**Authors:** N. Levernier, M. Dolgushev, O. Bénichou, R. Voituriez, T. Guérin

**Affiliations:** 1 NCCR Chemical Biology, Departments of Biochemistry and Theoretical Physics, University of Geneva, Geneva, Switzerland; 2 Laboratoire de Physique Théorique de la Matière Condensée, CNRS/Sorbonne Université, 4 Place Jussieu, 75005 Paris, France; 3 Laboratoire Jean Perrin, CNRS/Sorbonne Université, 4 Place Jussieu, 75005 Paris, France; 4 Laboratoire Ondes et Matière d’Aquitaine, University of Bordeaux, Unité Mixte de Recherche 5798, CNRS, F-33400 Talence, France

**Keywords:** Chemical physics, Statistical physics

## Abstract

For many stochastic processes, the probability $S(t)$ of not-having reached a target in unbounded space up to time $t$ follows a slow algebraic decay at long times, $S(t) \sim {S}_{0}/{t}^{\theta }$. This is typically the case of symmetric compact (i.e. recurrent) random walks. While the persistence exponent $\theta$ has been studied at length, the prefactor ${S}_{0}$, which is quantitatively essential, remains poorly characterized, especially for non-Markovian processes. Here we derive explicit expressions for ${S}_{0}$ for a compact random walk in unbounded space by establishing an analytic relation with the mean first-passage time of the same random walk in a large confining volume. Our analytical results for ${S}_{0}$ are in good agreement with numerical simulations, even for strongly correlated processes such as Fractional Brownian Motion, and thus provide a refined understanding of the statistics of longest first-passage events in unbounded space.

## Abstract (web summary)

The survival probability of a random walker is the probability that a particular target has not been reached by time *t*. Here the authors produce a formula for the prefactor involved in the expression of the survival probability which is shown to hold for both Markovian and non-Markovian processes.

## Introduction

In order to determine the time it takes for a random walker to find a target, or the probability that a stochastic signal has not reached a threshold up to time $t$, it is required to analyse the first-passage time (FPT) statistics. This has attracted considerable attention from physicists and mathematicians in the last decades^1–6^ notably because of the relevance of FPT related quantities in contexts as varied as diffusion controlled reactions, finance, search processes, or biophysics^7,8^.

A single-target first-passage problem is entirely characterized by the so-called “survival probability” $S(t)$ (the probability that the target has not been reached up to time $t$), or equivalently by the FPT distribution $F(t)=-{\partial }_{t}S(t)$. For a symmetric random walk in a confined domain, the mean FPT is in general finite and has been studied at length. This led recently to explicit results for broad classes of stochastic processes^2,9–12^. The opposite case of unconfined random walks is drastically different. In this case, either the walker has a finite probability of never finding the target (non-compact random walks), or it reaches it with probability one (compact random walk) and the survival probability decays algebraically with time, $S(t) \sim {S}_{0}/{t}^{\theta }$, with $\theta$ the persistence exponent that does not depend on the initial distance to the target. In this case the mean FPT is often infinite so that the relevant observable to quantify FPT statistics is the long-time algebraic decay of the probability $S(t)$ that the target has not been reached up to $t$. This, additional to the fact that $\theta$ can be non-trivial for non-Markovian random walks, has triggered a considerable amount of work to characterize the persistence exponent $\theta$ in a wide number of models of non-equilibrium statistical mechanics. Indeed, $S(t)$ is an essential observable to quantify the kinetics of transport controlled reactions and the dynamics of coarsening in phase transitions in general^13,14^.

However, if one aims to evaluate the time $t$ to wait for observing a first-passage event with a given likelihood, or to determine the dependence of the survival probability on the initial distance to the target, one needs to know the prefactor ${S}_{0}$, which turns out to be much less characterized than the persistence exponent $\theta$. Even for Markovian random walks this problem is not trivial^15^, as exemplified by recent studies for one-dimensional Levy flights^16^, while only scaling relations for ${S}_{0}$ (with the initial distance to the target) are known^17^ in fractal domains. However, if the dynamics of the random walker results from interactions with other degrees of freedom, the process becomes non-Markovian and the determination of ${S}_{0}$ becomes much more involved^18^. In this case, the only explicit results are derived from perturbation expansion around Markovian processes^19,20^, or have been obtained for particular processes such as “run and tumble” motion (driven by telegraphic noise^21^) or the random acceleration process^22^. For long-range correlated processes, such as fractional Brownian Motion, the existence of ${S}_{0}$ is not even established rigourously^15,23^, and it has been found that straightforward adaptation of Markovian methods can lead to order-of-magnitude overestimations of ${S}_{0}$ and even to erroneous scalings^24^.

In this article, we rely on a non-perturbative strategy to determine ${S}_{0}$, which is of crucial interest to quantify the statistics of long FPT events. Our main result is a relation between the prefactor ${S}_{0}$ in the long-time survival probability in free space and the mean FPT for the same process in a large confining volume. Our formula thus shows how to make use of the wealth of explicit results obtained recently on first-passage properties in confinement^2,9,10,25^ to determine the decay of the free-space survival probability. This formula is shown to be robust and holds for Markovian or non-Markovian processes with stationary increments, that are scale invariant at long times with diverging moments of the position, in one or higher spatial dimensions, and also for processes displaying transient aging (i.e., processes with finite memory time, whose initial state is not stationary, see below). This theory is confirmed by simulations for a variety of stochastic processes, including highly correlated ones such as Fractional Brownian Motion.

## Results

### Markovian case

**Fig. 1:**
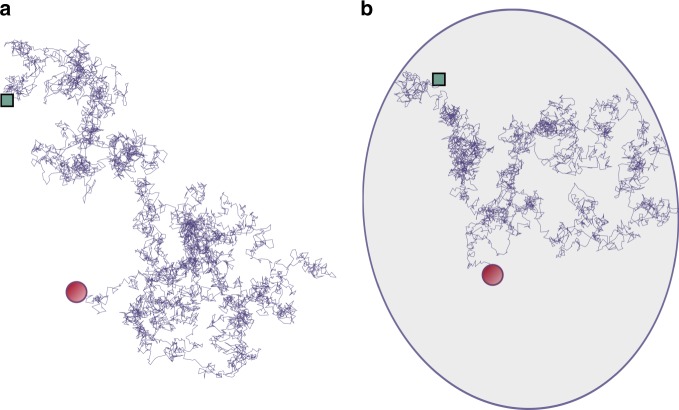
First-passage problem with or without confinement. Two first-passage problems in which a random walker starting from a given site (green square) reaches a target (red disk) at the end of a stochastic trajectory: **a** in free space, **b** in a confined reflecting domain. Sample trajectories for fractional Brownian motion ($H=0.45$) are shown

We consider a symmetric random walker of position ${\bf{r}}(t)$ moving on an infinite discrete lattice (potentially fractal) of dimension ${d}_{f}$ (see Fig. 1a for the continuous space counterpart) in continuous time $t$, in absence of external field. The initial position is ${{\bf{r}}}_{0}$. We assume that the increments are stationary (no aging), which means in particular that $\sigma (t,\tau )\equiv \langle | {\bf{r}}(t+\tau )-{\bf{r}}(t){| }^{2}\rangle$ is independent of the elapsed time $t$. Note that in the case of fractal spaces, we use the standard “chemical” distance defined as the minimal number of steps to link two points on the lattice. We define the walk dimension ${d}_{w}$ such that $\sigma (t,\tau )\propto {\tau }^{2/{d}_{w}}$ for $\tau \to \infty$. Note that (i) this scale invariance is assumed only at long times, and that (ii) it implies that all even moments of the position diverge with time. We assume ${d}_{w} > {d}_{f}$ so that the process is compact^26,27^ (and eventually reaches any point with probability one). We also introduce the Hurst exponent $H=1/{d}_{w}$.

We first consider the case of Markovian (memoryless) random walks. One can then define a propagator $p({\bf{r}},t| {{\bf{r}}}_{0})$, which represents the probability to observe the walker at site ${\bf{r}}$ at time $t$ given that it started at ${{\bf{r}}}_{0}$ at initial time. Note that $p$ is defined in absence of target. We now add an absorbing target at site ${\bf{r}}=0$ (different from ${{\bf{r}}}_{0}$). We start our analysis with the standard renewal equation^1,18,28^:

1 $$p({\bf{0}},t| {{\bf{r}}}_{0})={\int _{0}^{t}}d\tau F(\tau ;{{\bf{r}}}_{0})p({\bf{0}},t-\tau | {\bf{0}}),$$

which relates the propagator $p$ to the FPT distribution $F$ that depends on ${{\bf{r}}}_{0}$. This equation is obtained by partitioning over the FPT to the target, and can be rewritten in Laplace space as

2 $$\widetilde{p}({\bf{0}},s| {{\bf{r}}}_{0})=\widetilde{F}(s;{{\bf{r}}}_{0})\widetilde{p}({\bf{0}},s| {\bf{0}}),$$

where $\widetilde{F}(s)={\int }_{\!0}^{\infty }dtF(t){e}^{-st}$ stands for the Laplace transform of $F(t)$. Here, we only focus on the long-time behavior of $F(t)$, that can be obtained by expanding Eq. (2) for small $s$. Scale invariance at long times implies^27^ that for any site ${\bf{r}}$

3 $$p({\bf{0}},t| {\bf{r}})\mathop{ \sim }\limits_{t\to \infty }K/{t}^{{d}_{f}/{d}_{w}},$$

where the notation “$\sim$” represents asymptotic equivalence, and $K$ is a positive coefficient. Note that $K$ is known to be position independent and is well characterized (at least numerically) for a large class of stochastic processes, including diffusion in a wide class of fractals^17^, ^29–31^. We find that the small-$s$ behavior of the propagator is

4 $$\frac{K\ \Gamma (1-\frac{{d}_{f}}{{d}_{w}})}{{s}^{1-{d}_{f}/{d}_{w}}}-\widetilde{p}({\bf{0}},s| {\bf{r}})\mathop{ \sim }\limits_{s\to 0}{\int _{0}^{\infty }}dt\left[\frac{K}{{t}^{{d}_{f}/{d}_{w}}}-p({\bf{0}},t| {\bf{r}})\right],$$

where $\Gamma (\cdot )$ is the Gamma function. Eqs. (2) and (4) (written for ${\bf{r}}={\bf{0}}$ and ${\bf{r}}={{\bf{r}}}_{0}$) lead to

5 $$1-\widetilde{F}(s;{{\bf{r}}}_{0})\mathop{ \sim }\limits_{s\to 0}{\int _{0}^{\infty }}dt\left[p({\bf{0}},t| {\bf{0}})-p({\bf{0}},t| {{\bf{r}}}_{0})\right]\frac{{s}^{1-{d}_{f}/{d}_{w}}}{K\ \Gamma \left(1-\frac{{d}_{f}}{{d}_{w}}\right)}.$$

Taking the inverse Laplace transform (and using $F(t)=-\dot{S}$) leads to $S(t) \sim {S}_{0}/{t}^{\theta }$ with $\theta =1-{d}_{f}/{d}_{w}$ (as found in ref. ^17^), and to

6 $${S}_{0}=\frac{\sin (\pi {d}_{f}/{d}_{w})}{K\pi }{\int _{0}^{\infty }}dt\left[p({\bf{0}},t| {\bf{0}})-p({\bf{0}},t| {{\bf{r}}}_{0})\right].$$

This expression is exact and characterizes the decay of the survival probability of unconfined scale invariant Markovian random walks.

We now consider the target search problem for the same random walk, with the only difference that it takes place in a confining volume $V$ (that is equal to the number of sites $N$ in our discrete formulation) (see Fig. 1b). For this problem, the mean FPT $\langle {\bf{T}}\rangle$ is in general finite and it is known that it scales linearly with the volume and reads^2,9^

7 $$\frac{\langle {\bf{T}}\rangle }{V}\mathop{ \sim }\limits_{V\to \infty }{\int _{0}^{\infty }}dt\left[p({\bf{0}},t| {\bf{0}})-p({\bf{0}},t| {{\bf{r}}}_{0})\right].$$

We recognize in the above expression the time integral of propagators appearing in Eq. (6), leading to

8 $${S}_{0}=\frac{\sin (\pi {d}_{f}/{d}_{w})}{\pi \ K}\overline{T},\,\text{with}\,\overline{T}=\mathop{\mathrm{lim}}\limits_{V\to \infty }\langle {\bf{T}}\rangle /V.$$

Hence, for compact Markovian random walks, we have identified a proportionality relation between the prefactor ${S}_{0}$ that characterizes the long-time survival probability in free space and the rescaled mean FPT to the target in unconfined space. The proportionality coefficient involves the walk dimension ${d}_{w}$ and the coefficient $K$ which characterizes the long-time decay of the propagator (see Eq. (3)). Formula (8) is the key result of this paper. As we proceed to show, it is very robust and is not limited to Markovian walks.

As a first application, consider the case of scale invariant Markovian random walks (such as diffusion on fractals), for which it was shown^32^ that $\overline{T}\simeq {r}_{0}^{{d}_{w}-{d}_{f}}$, where the mean waiting time on a given site is taken as unity, and ${r}_{0}$ is the initial source-target (chemical) distance. Inserting this formula into Eq. (8) thus leads to

9 $${S}_{0}\simeq \frac{\sin (\pi {d}_{f}/{d}_{w}){r}_{0}^{{d}_{w}-{d}_{f}}}{\pi \ K}.$$

**Fig. 2:**
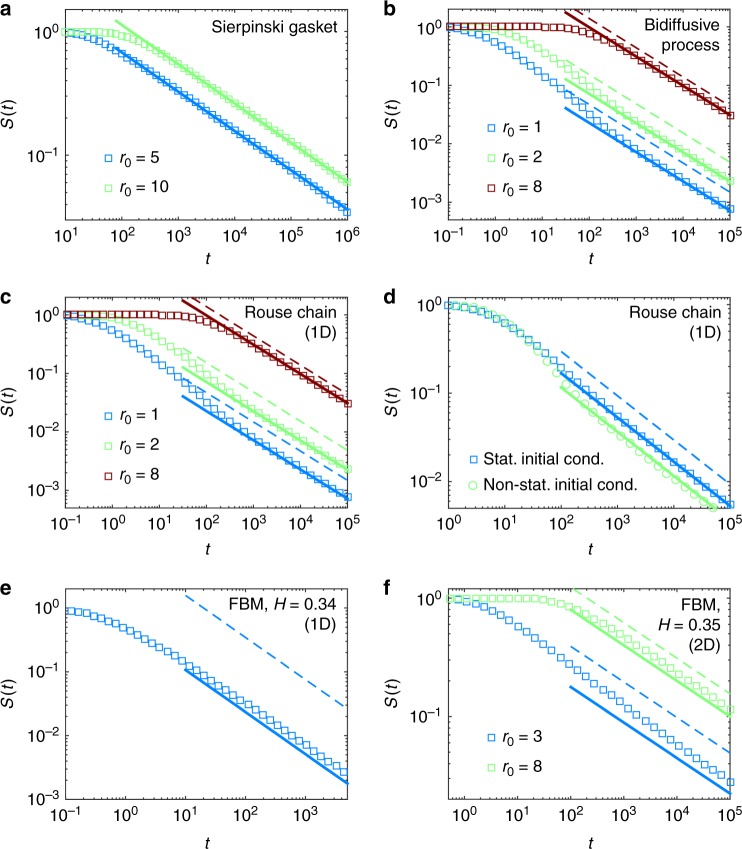
Survival probability $S(t)$ for various stochastic processes. In all graphs, symbols are the results of stochastic simulations (detailed in SI), continuous lines give the theoretical predictions (Eq. (18)), and dashed line represent the predictions of the pseudo-Markovian approximation (The pseudo-Markovian approximation, which is similar to the Wilemski–Fixman approximation for the polymer cyclization kinetics problem, consists in using effective propagators in Eq. (18), i.e $p({\bf{x}},t| {{\bf{x}}}_{0})={e}^{-{({\bf{x}}-{{\bf{x}}}_{0})}^{2}/2\psi (t)}/{(2\pi \psi (t))}^{d/2}$.). **a** $S(t)$ for a random walk on the Sierpinski gasket for two values of the initial (chemical) source-target distance. Here, ${d}_{f}=\mathrm{ln}3/\mathrm{ln}2$, ${d}_{w}=\mathrm{ln}5/\mathrm{ln}2$, and $K\simeq 0.30$^31^. Simulations are shown for a fractal of generation $11$. Continuous lines are the predictions of Eq. (9). **b** $S(t)$ for a one-dimensional “bidiffusive” Gaussian process of MSD $\psi (t)=t+30(1-{e}^{-t})$. **c** $S(t)$ for a one-dimensional Rouse chain with $N=20$ monomers, for various source-to-target distance ${r}_{0}$ (indicated in the legend in units of the monomer length). **d** $S(t)$ for the same system with $N=15$ and ${r}_{0}=3$, comparing stationary initial conditions (the other monomers being initially at equilibrium) or non-stationary ones (for which all monomers start at the same position ${r}_{0}$). **e** $S(t)$ for a one-dimensional FBM of MSD $\psi (t)={t}^{2H}$ with Hurst exponent $H=0.34$. **f** Two-dimensional FBM of MSD $\psi (t)={t}^{2H}$ in each spatial direction with $H=0.35$. The target is a disk of radius $a=1$ and ${r}_{0}$ is the distance to the target center. For (**b**), (**c**), (**d**), (**e**), and (**f**), the continuous lines represent our predictions (Eq. (18)), in which $\overline{T}$ is calculated by using the theories of refs. ^12,25,48^; in (**b**) and (**c**) the only hypothesis to predict $\overline{T}$ is that the distribution of supplementary degrees of freedoms at the FPT is Gaussian, in (**e**) and (**f**) we use the additional “stationary covariance” approximation. In (**d**), for non-stationary initial conditions, $\overline{T}$ is measured in simulations in confined space. A table that compares the values of ${S}_{0}$ in the theory and in the simulations is given in SI

In this case, we thus recover the scaling result of ref. ^17^ but in addition obtain the value of the prefactor. We have checked this relation for the Sierpinski gasket: simulation results are shown in Fig. 2a. The long-time persistence is perfectly described by our formula without any fitting parameter for different source-target distances, confirming the validity of our approach (see SI for other examples).

As a second application, we can consider the one-dimensional Lévy stable process of index $\alpha$, which is defined as the only Markovian process whose jump distribution is given by $p(\Delta x,t)=1/(2\pi ){\int }_{-\infty }^{\infty }{e}^{i\Delta x.k-t| k{| }^{\alpha }}dk$. This process, defined in continuous space, is the continuous time limit of the Lévy Flight with same index $\alpha$. Its walk dimension is ${d}_{w}=\alpha$ and it is compact for $\alpha > 1$, so that the first passage to a point target is well defined (note that we consider here the first arrival at the target, and not the first crossing event^33,34^). For such a process, the prefactor ${S}_{0}$ for an unconfined random walk starting at a distance ${r}_{0}$ from the target has been shown to be ${S}_{0}=\alpha \sin (\pi \alpha /2)\sin (\pi /\alpha )\Gamma (2-\alpha ) {r}_{0}^{\alpha -1}/(\pi \Gamma (1/\alpha )(\alpha -1))$^35^. By computing the rescaled MFPT in confinement with Eq. (7), one can check that the relation (8), which can be readily generalized to continuous space, is still verified for this process.

### Extension to non-Markovian processes

We now relax the Markov property and generalize our theory to the case of non-Markovian processes, i.e., displaying memory. In the following, we argue that the relation (8) yields much more accurate results for ${S}_{0}$ than Markov approximations; it is exact for processes with finite memory time, and is very precise (even though not exact) for strongly correlated processes such as the Fractional Brownian Motion. As the mean FPT in confinement has recently been characterized for non-Markovian Gaussian processes^25^, this equation (8) provides a means to estimate $S(t)$ at long times, beyond persistence exponents, for a wide class of random walks with memory.

For simplicity, we consider one-dimensional processes and we switch to continuous space description. The stochastic trajectories $x(t)$ are assumed to be continuous but non-smooth (the method in fact also applies to compact and not continuous processes, such as $1d$ Levy stable processes of index $\alpha > 1$ as discussed above), mathematically meaning that $\langle {\dot{x}}^{2}\rangle =\infty$ and physically corresponding to very rough trajectories, similar to those of Brownian motion. We assume that the increments of the walk are stationary (meaning that there is no aging, even transient (In particular, the case of continuous time random walks (CTRWs) is not directly covered by our analysis; persistence exponents and prefactors for CTRWs can be obtained from the subordination principle)). This hypothesis is known to have two consequences: (i) the persistence exponent for the unconfined problem is exactly given by $\theta =1-1/{d}_{w}$^14,15,23,36–38^; (ii) the mean FPT for the confined problem varies linearly with the confinement volume $V$, so that $\overline{T}$ is finite and has been identified as^25^:

10 $$\overline{T}={\int _{0}^{\infty }}dt\ [{p}_{\pi }(0,t)-p(0,t)].$$

Here, $p(0,t)$ is the probability density of $x=0$ at a time $t$ after the initial state (where $x(0)={r}_{0}$), and ${p}_{\pi }(0,t)$ denotes the probability density of finding the walker on the target at a time $t$ after the first-passage:

11 $${p}_{\pi }(0,t)={\int _{0}^{\infty }}d\tau \ p(0,t+\tau | {\rm{FPT}}=\tau )F(\tau ;{r}_{0}),$$

where $p(0,t+\tau | {\rm{FPT}}=\tau )$ is the probability density of $x=0$ at time $t+\tau$, given that the FPT is $\tau$.

The starting point to relate $\overline{T}$ to ${S}_{0}$ consists in writing the generalization of Eq. (1) to non-smooth non-Markovian processes:

12 $$p(0,t)={\int _{0}^{t}}d\tau F(\tau ;{r}_{0})p(0,t| {\rm{FPT}}=\tau ).$$

To proceed further, we insert into Eq. (10) the expressions (11) and (12) of ${p}_{\pi }(0,t)$ and $p(0,t)$:

13 $$\overline{T} =	 {\int _{0}^{\infty }}dt\ \left[{\int _{0}^{\infty }}d\tau \ p(0,t+\tau \ | \ \text{FPT}=\tau )\ F(\tau ;{r}_{0})\right. \\ 	\left. -{\int _{0}^{t}}d\tau \ p(0,t\ | \ \text{FPT}=\tau )\ F(\tau ;{r}_{0})\right].$$

To avoid diverging integrals in the change of variables $t=u+\tau$, we replace ${\int }_{\!0}^{\infty }dt(...)$ by $\mathop{\mathrm{lim}}\limits_{A\to \infty }\ \ {\int }_{\!0}^{A}dt(...)$, so that

14 $$\overline{T}=\mathop{\mathrm{lim}}\limits_{A\to \infty }{\int _{0}^{A}}dt\ {\int _{A-t}^{\infty }}d\tau F(\tau ;{r}_{0})p(0,t+\tau | \text{FPT}=\tau ).$$

Setting $t=uA$ and $\tau =vA$, we note that when $A\to \infty$, only the large time behavior are involved in these integrals, where one can use the asymptotics $F(Av;{r}_{0}) \sim {S}_{0}\theta /{(Av)}^{1+\theta }$ and

15 $$p(0,(u+v)A| \text{FPT}=vA)\mathop{ \sim }\limits_{A\to \infty }\frac{KG(u/v)}{{(Au)}^{1/{d}_{w}}},$$

which is a form imposed by dimensional analysis. As previously, $K$ is the constant which characterizes the long-time behavior of the one point probability distribution function (i.e., $p(x,t)\mathop{ \sim }\limits_{t\to \infty }K/{t}^{1/{d}_{w}}$), $G$ is a scaling function, with $G(\infty )=1$, that does not depend on the geometrical parameters of the problem. Inserting these asymptotic behaviors into Eq. (14), we get:

16 $$\overline{T}=\mathop{\mathrm{lim}}\limits_{A\to \infty }{A}^{1-\theta -1/{d}_{w}}\ K\ {\int _{0}^{1}}du\ {\int _{1-u}^{\infty }}dv\ \frac{\theta \ {S}_{0}}{{v}^{\theta +1}}\frac{G(u/v)}{{u}^{1/{d}_{w}}}.$$

The fact that the above integral exists and is finite leads to the (known) relation $\theta =1-1/{d}_{w}$. This finally leads to the exact relation:

17 $${S}_{0}=\frac{\overline{T}}{K(1-1/{d}_{w})}{\left({\int _{0}^{1}}du{\int _{1-u}^{\infty }}dv\frac{G(u/v)}{{u}^{1/{d}_{w}}{v}^{2-1/{d}_{w}}}\right)}^{-1}.$$

We stress that the dependency of ${S}_{0}$ on the source-to-target distance, even when not trivial, is entirely contained in the term $\overline{T}$. Indeed, the scaling function $G$ depends only on the large scale properties of the random walk and not on the geometrical parameters.

While the exact determination of $G$ is a challenging task, the following decoupling approximation turns out to be very accurate. In this approximation, the return probability to the target at a time $t$ after the first-passage time is independent of the actual value of the FPT, which leads to $p(0,t+\tau | \text{FPT}=\tau )\simeq {p}_{\pi }(t)$ for self-consistence reason. Within this decoupling approximation, $G\simeq 1$ and we obtain

18 $${S}_{0}\simeq \frac{\sin (\pi /{d}_{w})}{K\pi }\overline{T},$$

which generalizes Eq. (8) to non-Markovian processes. We now comment on the validity of this key relation.

First, we stress that Eq. (18) is exact for processes with finite memory time (i.e. for which the correlation function of increments decays exponentially at long times). This comes from the very definition of the function $G$, which involves only large time scales in Eq. (15), over which this finite memory time becomes irrelevant. This case is illustrated here by considering a Gaussian process whose Mean Square Displacement function $\psi (t)=\langle {[x(t+\tau )-x(\tau )]}^{2}\rangle$ is given by $\psi (t)=Dt+B(1-{e}^{-\lambda t})$. This “bidiffusive” process involves two diffusive behaviors at long and short time scales, and presents only one relaxation time ${\lambda }^{-1}$. This is typically relevant to tracers moving in viscoelastic Maxwell fluids^39^, nematics^40^, or solutions of non-adsorbing polymers^41^. We also consider the effect of multiple relaxation times with the case that $x(t)$ is the position of the first monomer of a flexible polymer chain with $N$ monomers, in the most simple (Rouse, bead-spring) polymer model. We use recently obtained estimates of $\overline{T}$ in ref. ^25^ to obtain estimates of ${S}_{0}$ through Eq. (18) and compare with numerical simulations in Fig. 2b, c. We also compare with a pseudo-Markovian approximation (using Eq. (6) with effective “propagators” (The pseudo-Markovian approximation, which is similar to the Wilemski–Fixman approximation for the polymer cyclization kinetics problem, consists in using effective propagators in Eq. (18), i.e., $p({\bf{x}},t| {{\bf{x}}}_{0})={e}^{-{({\bf{x}}-{{\bf{x}}}_{0})}^{2}/2\psi (t)}/{(2\pi \psi (t))}^{d/2}$.)). Our prediction for ${S}_{0}$ is in good agreement with numerical simulations, and shows that even if the memory time is finite, memory effects are strong.

Second, it is showed in SI that Eq. (18) is also exact at first order in $\varepsilon =H-1/2$ for the fractional Brownian motion (FBM), which is an emblematic example of processes with infinite memory time. The FBM is used in fields as varied as hydrology^42^, finance^43^, and biophysics^44,45^. This Gaussian process is characterized by $\left\langle {[x(t+\tau )-x(t)]}^{2}\right\rangle =\kappa {\tau }^{2H}$, with $0 < H < 1$.

Third, in the strongly non-Markovian regime, where $\varepsilon$ cannot be considered as small, it turns out that Eq. (18) provides a very accurate approximation (Fig. 2e) of ${S}_{0}$, which takes the explicit form

19 $${S}_{0}={\beta }_{H}\sin (\pi H)\sqrt{\frac{2}{\pi }}{\left(\frac{{r}_{0}}{{\kappa }^{1/2}}\right)}^{\frac{1}{H}-1}$$

where ${\beta }_{H}$ is a function of $H$ analyzed in ref. ^25^. It can indeed be seen in Fig. 2e that Eq. (18) correctly predicts the long-time behavior of $S(t)$ when $H=0.34$. For this value, non-Markovian effects are strong, as can be seen by comparing with the prediction of the pseudo-Markovian approximation, which is wrong by more than one order of magnitude (Fig. 2e, dashed line). The value of ${S}_{0}$ is slightly underestimated in the decoupling approximation, but can be made more precise by evaluating the scaling function $G$ (see SI).

Furthermore, our approach also holds in dimension higher than one, even for strongly correlated non-Markovian processes. Indeed, the $d-$dimensional version of Eq. (18) (i.e., Eq. (8)) correctly predicts (but slightly underestimates) the value of ${S}_{0}$ for an example of two-dimensional FBM (Fig. 2f). In this example, the target radius $a$ is not zero even if the $a\to 0$ limit is well defined for compact processes; the dependence of ${S}_{0}$ on the target radius is predicted to be the same as that of $\overline{T}$, which is available in the non-Markovian theory of ref. ^25^. Finally, in the case of processes with finite memory, we find that Eq. (18) also holds for non-stationary initial conditions. This is illustrated by considering the case of a flexible phantom polymer for which all monomers are placed initially at ${r}_{0}$ (instead of having the shape of a random equilibrium coil for stationary initial conditions). This non-stationary initial condition induces transiently aging dynamics, and ${S}_{0}$ is changed with respect to the case of stationary initial conditions, but is still predicted correctly by Eq. (18) (see Fig. 2d).

Finally, let us mention the case of the one-dimensional run and tumble process, where a particle switches between phases of constant velocities $\pm v$ with rate $\alpha$. This process is smooth and is a priori not covered by our analysis. However, our relation (18) between ${S}_{0}$ and $T/V$ is still exact, as is made clear by comparing the results for the mean FPT in confinement^21^ and in semi-infinite space^46^. This agreement holds even for non-stationary initial conditions, where the probability $p$ that the initial velocity is positive differs from $1/2$: in this case, one can obtain ${S}_{0}=({r}_{0}+pv/\alpha )\sqrt{2\alpha /(\pi {v}^{2})}=\overline{T}/(K\pi )$, with $K=\sqrt{\alpha /(2\pi {v}^{2})}$, and we can check that our relation still holds^21,46^. Furthermore, it also holds in the case of partially reflecting targets, as can be deduced from the results of ref. ^47^. This suggests that our analysis can be extended to smooth non-Markovian processes with partial absorption as well.

## Discussion

The determination of the survival probability $S(t)$, and in particular its dependence on the initial distance to the target, requires the knowledge of its prefactor ${S}_{0}$, which has remained an elusive quantity up to now. In this article, we have bridged this gap by identifying a general relation between the long-time persistence and the mean FPT in confinement. The latter can be calculated with various recently introduced methods, for a large class of Markovian^2,10,11^ and non-Markovian random walks^25^. Our theory holds for compact, unbiased walks with stationary increments that are scale invariant at long times (without confinement), with moments of the position that diverge with time. Our main result is Eq. (8), which is exact for both Markovian processes (such as diffusion in fractals) and for non-Markovian processes with finite memory time (for which memory effects are nevertheless quantitatively non-negligible). For long-ranged correlated processes such as FBM our formula provides a good approximation of ${S}_{0}$ in one or higher dimensions, and is found to be exact at first order in a perturbation expansion around Brownian motion. Together, our results thus improve our understanding of the impact of memory on the statistics of long first-passage events.

## Supplementary information

Supplementary Information

## Data Availability

The numerical data presented in Fig. 2 are available from the corresponding author on reasonable request.

## Code availability

The code that generated these data are available from the corresponding author on reasonable request.

## References

[1] Redner S (2001). A Guide to First-Passage Processes.

[2] Condamin S, Bénichou O, Tejedor V, Voituriez R, Klafter J (2007). First-passage times in complex scale-invariant media. Nature.

[3] Pal A, Reuveni S (2017). First passage under restart. Phys. Rev. Lett..

[4] Grebenkov DS (2016). Universal formula for the mean first passage time in planar domains. Phys. Rev. Lett..

[5] Bénichou O, Grebenkov D, Levitz P, Loverdo C, Voituriez R (2010). Optimal reaction time for surface-mediated diffusion. Phys. Rev. Lett..

[6] Vaccario G, Antoine C, Talbot J (2015). First-passage times in d-dimensional heterogeneous media. Phys. Rev. Lett..

[7] Metzler, R., Redner, S. & Oshanin, G. *First-Passage Phenomena and Their Applications* (World Scientific, 2014).

[8] Berg OG, vonHippel PH (1985). Diffusion-controlled macromolecular interactions. Annu. Rev. Biophys. Biophys. Chem..

[9] Condamin S, Bénichou O, Moreau M (2005). First-passage times for random walks in bounded domains. Phys. Rev. Lett..

[10] Bénichou O, Voituriez R (2008). Narrow-escape time problem: time needed for a particle to exit a confining domain through a small window. Phys. Rev. Lett..

[11] Schuss Z, Singer A, Holcman D (2007). The narrow escape problem for diffusion in cellular microdomains. Proc. Natl Acad. Sci. USA.

[12] Guérin T, Bénichou O, Voituriez R (2012). Non-Markovian polymer reaction kinetics. Nat. Chem..

[13] Majumdar, S. N. Persistence in nonequilibrium systems. *Curr. Sci.* **77**, 370–375 (1999).

[14] Bray AJ, Majumdar SN, Schehr G (2013). Persistence and first-passage properties in nonequilibrium systems. Adv. Phys.

[15] Aurzada, F. & Simon, T. in *Lévy Matters**V* 183–224 (Springer, 2015).

[16] Majumdar SN, Mounaix P, Schehr G (2017). Survival probability of random walks and Lévy flights on a semi-infinite line. J. Phys. A: Math. Theor.

[17] Meroz Y, Sokolov IM, Klafter J (2011). Distribution of first-passage times to specific targets on compactly explored fractal structures. Phys. Rev. E.

[18] Van Kampen, N. *Stochastic Processes in Physics and Chemistry* 3rd edn (Amsterdam, 2007).

[19] Delorme M, Wiese KJ (2015). Maximum of a fractional Brownian motion: analytic results from perturbation theory. Phys. Rev. Lett..

[20] Delorme M, Wiese KJ (2016). Perturbative expansion for the maximum of fractional Brownian motion. Phys. Rev. E.

[21] Masoliver J, Lindenberg K, West BJ (1986). First-passage times for non-Markovian processes: correlated impacts on a free process. Phys. Rev. A.

[22] Burkhardt TW (1993). Semiflexible polymer in the half plane and statistics of the integral of a Brownian curve. J. Phys. A: Math. Gen..

[23] Aurzada F (2011). On the one-sided exit problem for fractional Brownian motion. Electron. Commun. Probab..

[24] Sanders LP, Ambjörnsson T (2012). First passage times for a tracer particle in single file diffusion and fractional Brownian motion. J. Chem. Phys..

[25] Guérin T, Levernier N, Bénichou O, Voituriez R (2016). Mean first-passage times of non-Markovian random walkers in confinement. Nature.

[26] DeGennes P-G (1982). Kinetics of diffusion-controlled processes in dense polymer systems. 1. Non-entangled regimes. J. Chem. Phys..

[27] benAvraham D, Havlin S (2000). Diffusion and Reactions in Fractals and Disordered Systems.

[28] Hughes, B. D. *Random Walks and Random Environments* (Oxford Science Publications, 1995).

[29] Grabner PJ, Woess W (1997). Functional iterations and periodic oscillations for simple random walk on the Sierpiński graph. Stoch. Proc. Their Appl..

[30] Krön B, Teufl E (2004). Asymptotics of the transition probabilities of the simple random walk on self-similar graphs. Trans. Am. Math. Soc.

[31] Weber S, Klafter J, Blumen A (2010). Random walks on Sierpinski gaskets of different dimensions. Phys. Rev. E.

[32] Bénichou O, Meyer B, Tejedor V, Voituriez R (2008). Zero constant formula for first-passage observables in bounded domains. Phys. Rev. Lett..

[33] Chechkin AV, Metzler R, Gonchar VY, Klafter J, Tanatarov LV (2003). First passage and arrival time densities for Lévy flights and the failure of the method of images. J. Phys. A: Math. Gen..

[34] Tejedor V, Bénichou O, Metzler R, Voituriez R (2011). Residual mean first-passage time for jump processes: theory and applications to Lévy flights and fractional Brownian motion. J. Phys. A: Math. Theor.

[35] Blumenthal RM, Getoor RK, Ray DB (1961). On the distribution of first hits for the symmetric stable processes. Trans. Am. Math. Soc.

[36] Levernier N, Bénichou O, Guérin T, Voituriez R (2018). Universal first-passage statistics in aging media. Phys. Rev. E.

[37] Molchan G (1999). Maximum of a fractional Brownian motion: probabilities of small values. Commun. Math. Phys..

[38] Krug J (1997). Persistence exponents for fluctuating interfaces. Phys. Rev. E.

[39] Grimm M, Jeney S, Franosch T (2011). Brownian motion in a maxwell fluid. Soft Matter.

[40] Turiv T (2013). Effect of collective molecular reorientations on Brownian motion of colloids in nematic liquid crystal. Science.

[41] Ochab-Marcinek A, Hołyst R (2011). Scale-dependent diffusion of spheres in solutions of flexible and rigid polymers: mean square displacement and autocorrelation function for FCS and DLS measurements. Soft Matter.

[42] Mandelbrot BB, Wallis JR (1968). Noah, Joseph, and operational hydrology. Water Resour. Res..

[43] Cutland, N. J., Kopp, P. E. & Willinger, W. in *Seminar on Stochastic Analysis, Random Fields and Applications* 327–351 (Springer, 1995).

[44] Ernst D, Hellmann M, Köhler J, Weiss M (2012). Fractional Brownian motion in crowded fluids. Soft Matter.

[45] Burnecki K (2012). Universal algorithm for identification of fractional Brownian motion. A case of telomere subdiffusion. Biophys. J..

[46] Malakar K (2018). Steady state, relaxation and first-passage properties of a run-and-tumble particle in one-dimension. J. Stat. Mech..

[47] Angelani L, DiLeonardo R, Paoluzzi M (2014). First-passage time of run-and-tumble particles. Eur. Phys. J. E.

[48] Guérin T, Bénichou O, Voituriez R (2013). Reactive conformations and non-Markovian kinetics of a Rouse polymer searching for a target in confinement. Phys. Rev. E.

