# Supplementary material for: Survival probability of stochastic processes beyond persistence exponents
Source: Nat Commun. 2019 Jul 5;10:2990. doi: 10.1038/s41467-019-10841-6 (PMC6611868; doi:10.1038/s41467-019-10841-6)
Supplement: Supplementary file 1 — Supplementary Information [file 41467_2019_10841_MOESM1_ESM.pdf]

SUPPLEMENTARY INFORMATION
Survival probability of stochastic processes beyond persistence exponents

N. Levernier, M. Dolgushev, O. Bénichou, R. Voituriez, and T. Guérin

In this supplementary information, we provide:

1. details on simulations used to generate Fig. 2 in the main text (Supplementary Note 1),
2. a proof that our formulas, within the decoupling approximation, are exact in first order of perturbation around Brownian motion the for the fractional Brownian motion (Supplementary Note 2),
3. additional information on the scaling function G (Supplementary Note 3).

Supplementary Note 1: Details on numerical simulations

Here we give additional details on how the results presented on Fig. 2 (in the main text) are obtained.

Diffusion on fractal lattices [Fig.2(a) and Supplementary Figure 1]. We performed simulations of random walks on the Sierpinski gasket (SG) and the dual Sierpinski gasket (DSG). The generation g of the lattice was chosen to be high enough to consider that the search for the target occurs in infinite space (we used $g = 11$ for the SG and $g = 10$ for the DSG). For these fractals we have first randomly selected a pair of nodes having minimal chemical distance r_0 and then performed random walk simulation. The procedure was repeated between 10^5 and 10^6 times. For determination of the survival probability $S(t)$ we have used a cut-off of 10^6 steps. The mean-first passage time $\overline{T}$ was calculated from simulations without cut-off by performing random walks on fractals of lower generations (up to $g = 8$, so that, for selected distances r_0, $\overline{T}$ showed a saturation to a constant value). For the calculation of the prefactor S_0 we have used the behavior of the propagator, $p(\mathbf{r}, t) \sim K/t^{d_f/d_w}$. For SG and DSG the fractal and walk dimensions are $d_f = \ln 3/\ln 2$ and $d_w = \ln 5/\ln 2$, respectively. For SG the constant $K \approx 0.30$, see [1, 2], and for DSG $K \approx 0.37$, see [2]. Note that the constant K is the same for all distances r that are much smaller than the size of the fractal [3, 4].

Flexible (Rouse) chain [Fig.2(c) and (d)]. We consider the model in which $x(t) = x_1(t)$ is the position of the first monomer of a one dimensional Rouse chain of N monomers, whose dynamics is

$$\zeta \dot{x}_i = -k(x_{i+1} - 2x_i + x_{i-1}) + f_i(t) \qquad (1)$$

with $1 \le i \le N$, $\langle f_i(t) f_j(t')\rangle = 2\delta_{ij}\delta(t-t')k_B T\zeta$, and $x_0(t) = x_1(t)$, $x_{N+1}(t) = x_N(t)$ by convention [5]. In our numerical simulations we integrate numerically the Langevin equation, Supplementary Equation (1). Results are shown by setting the units so that $k = \zeta = k_B T = 1$. We consider two choices of initial condition: (i) stationary initial condition [Fig.2(c)], in which the whole chain is initially at equilibrium conditional to $x_1(0) = r_0$, and (ii) non-stationary initial condition [Fig.2(d)], in which the position of all monomers is set to r_0 at initial time. In case (ii), the dynamics displays transient aging, the non-stationary initial condition is forgotten if one waits more than the largest relaxation time of the chain $\sim N^2$. In Fig.2(c), $N = 20$ and the time step is $\Delta t = 2 \times 10^{-4}$, whereas in Fig.2(d) $N = 15$ and $\Delta t = 10^{-3}$.

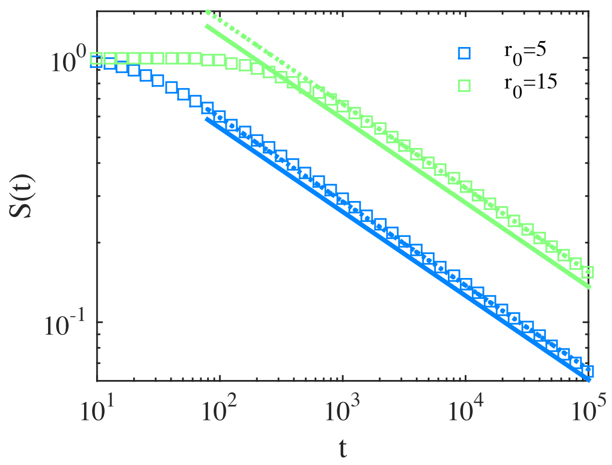

Supplementary Figure 1: Survival probability for diffusion on the dual Sierpinski gasket with one absorbing site for different source-to-target chemical distances r_0. Symbols: simulations. Continuous lines: explicit prediction Eq. (9). Dashed lines: exact prediction of Eq. (8), in which $\overline{T}$ is measured in simulations.

Fractional Brownian Motion [FBM, Fig. 2(e) and (f)]. The FBM is a symmetric Gaussian process with stationary increments with mean square displacement $\langle [x(t) - x(0)]^2\rangle = \kappa t^{2H}$. We simulate it with by using the circulant matrix algorithm [6–9], which exactly samples an FBM trajectory at times separated by a fixed time step Δt. We perform (i) one-dimensional simulations, where $x(0) = r_0 = 1$, and $\Delta t = 0.00006$ and (ii) two-dimensional simulations, where $\mathbf{x}(t) = (x(t), y(t))$ is a vector with $x(t)$ and $y(t)$ independent one dimensional FBMs, starting at $(r_0, 0)$, the absorbing region being a disk of radius $a = 1$, with a time step $\Delta t = 0.0015$. Results are shown in units with $\kappa = 1$. They are compared with the predictions for S_0 that use the link with

Process	r_0	S_0 in simulations	S_0 (our theory)	S_0 (Markovian Approx.)
Diffusion on SG	5	$2.92 < S_0 < 2.97$	2.918	-
	10	$4.9 < S_0 < 5$	4.863	-
Bidiffusive process	10	$2.1 < S_0 < 2.2$	2.11	4.7
	3	$0.17 < S_0 < 0.19$	0.159	0.575
	1	$0.032 < S_0 < 0.038$	0.033	0.827
Rouse (1D)	8	$9.6 < S_0 < 10$	9.82	13.88
	2	$0.72 < S_0 < 0.76$	0.713	1.53
	1	$0.23 < S_0 < 0.25$	0.228	0.469
FBM (1D, $H = 0.34$)	1	$0.65 < S_0 < 0.71$	0.49	7.2
FBM (2D, $H = 0.35$)	3	$0.85 < S_0 < 0.95$	0.71	1.56
	8	$3.5 < S_0 < 3.8$	3.17	4.92

Supplementary Table 1: Values of S_0 predicted by the theory versus simulation results corresponding to the processes shown in Fig. 2 in the main text. All units and parameters are given in the text of this SI [Supplementary Note 1].

$\overline{T}$ [Eq. (8)] and the method of Ref. [10] to compute the value of $\overline{T}$, within the "stationary covariance approximation". Specifically, predictions for $\overline{T}$ in 2D and $H = 0.35$ appear in Fig. 3c of Ref. [10]. For the one dimensional case, we use the prediction $\overline{T} = \beta_H x_0^{1/H-1}/\kappa^{2H}$, with $\beta_H \simeq 0.70$ [10] for $H = 0.34$.

Bidiffusive process [Fig. 2(b)]. We also simulate the "bidiffusive" process, that is the symmetric Gaussian process with stationary increments whose MSD function is $\psi(t) = Dt + B(1 - e^{-\lambda t})$. Results are shown in units for which $D = \lambda = 1$, and we use the value $B = 30$, and a time step $\Delta t = 1.5 \times 10^{-4}$. The bidiffusive process can be constructed by assuming that $x(t)$ is coupled to one additional degree of freedom for which the relaxation time is $1/\lambda$. Consider for example the Langevin system

$$\dot{a_1} = f_1(t),\ \dot{a_2} = -\lambda a_2 + f_2(t) \tag{2}$$

$$\langle f_i(t) f_j(t) \rangle = \delta_{ij}\delta(t - t'), \tag{3}$$

and define $x(t) = \sqrt{D}a_1(t) + \sqrt{B}a_2(t)$. Assuming that a_2 is initially at equilibrium and that x has initially a fixed value $x(0)$, we get the MSD of the bi-diffusive process $\psi(t) = \langle [x(t) - x(0)]^2 \rangle = Dt + B(1 - e^{-\lambda t})$.

Estimates for the prefactor S_0 are shown in Supplementary Table 1. Note that, in all our simulations, we begin with an arbitrary value of the time step and we decrease it by successive factors of 4 until we are convinced that the obtained results do not depend on the time step. We show the results for a sampling time where convergence has been reached.

Supplementary Note 2: Perturbation expansion for the prefactor S_0 for the Fractional Brownian Motion

Here we prove that our approach, with the decoupling approximation, provides an exact expression for S_0 at leading order in a perturbation expansion around Brownian motion for the fractional Brownian motion.

Consider a one dimensional fractional Brownian Motion (FBM), which is the symmetric Gaussian process $x(t)$ with stationary increments with $\langle [x(t) - x(0)]^2 \rangle = \kappa t^{2H}$, where κ is a generalized transport coefficient. If the process starts at x_0, we can state that the probability of not reaching the origin $S(t)$ behaves at

$$S(t) \underset{t\to\infty}{\sim} \alpha_H \left(\frac{x_0^{1/H}}{\kappa^{1/(2H)} t} \right)^{1-H}. \tag{4}$$

The above expression follows from dimensional analysis and from the fact the the persistence exponent is $\theta = 1 - H$. The problem is therefore reduced to the calculation of a single, dimensionless function of H. Here we characterize α at order $\varepsilon = H - 1/2$ at show that the decoupling approximation is exact at this order.

First, we derive α_H within our approach, and then use the comparison with the exact perturbation results of Refs. [11, 12]. In Ref. [10], it is found that the mean first passage time, rescaled by the confining volume, is exactly given by the following formula at order ε:

$$\overline{T} = \int_0^\infty dt \frac{1 - e^{-1/(2\pi t^{2H})}}{(2\pi t^{2H})^{1/2}} + \mathcal{O}(\varepsilon^2) \tag{5}$$

where we have used $x_0 = 1$ and $\kappa = 1$. Using the "decoupling approximation" of the main text, i.e. assuming $G \simeq 1$ for the scaling function G, our expression for S_0 is

$$S_0 = \frac{\sin(\pi H)}{\pi K}\overline{T}. \tag{6}$$

Here, K characterizes the long time behavior of the propagator and is identified from

$$p(x, t) \underset{t\to\infty}{\sim} \frac{1}{(2\pi t^{2H})^{1/2}}, \tag{7}$$

so that

$$K = 1/\sqrt{2\pi}. \tag{8}$$

Inserting this value into Supplementary Equation (6) and expanding at first order in ε leads to

$$\alpha_H \simeq \sqrt{\frac{2}{\pi}} \left[1 - 2\varepsilon(1 - \ln 2 - \gamma_e)\right], \tag{9}$$

where $\gamma_e = 0.577...$ is the Euler-Mascheroni constant. Here we remind that the only uncontrolled approximation in the above result is the use of the "decoupling approximation".

Let us now consider the exact perturbative results of Refs. [11, 12]. There it is shown that the survival probability reads

$$S(t, x_0) = \int_0^{x_0/\sqrt{2}t^H} f_H(y)\, dy. \quad (10)$$

Here the function f_H reads

$$f_H(y) = \sqrt{\frac{2}{\pi}} e^{-y^2/2} \left[1 + \varepsilon[W(y) + C_0] + \mathcal{O}(\varepsilon^2)\right], \quad (11)$$

where W is:

$$W(y) = {}_2F_2\left(1, 1; \frac{5}{2}, 3; \frac{y^2}{2}\right) \frac{y^4}{6} - 3y^2 + \sqrt{2\pi} e^{y^2/2} y + \pi(1 - y^2)\mathrm{erfi}\left(\frac{y}{\sqrt{2}}\right) + (y^2 - 2)[\gamma_E + \ln(2y^2)], \quad (12)$$

[see Eq. (17) of Ref. [11])]. The constant C_0 is deduced from the fact that $S(t = 0) = 1$. Using Supplementary Equation (10), this condition implies that $\int_0^\infty dy f(y) = 0$, which is satisfied at order ε when

$$C_0 = -\int_0^\infty dy \sqrt{\frac{2}{\pi}} e^{-y^2/2} W(y) = -2, \quad (13)$$

where the last equality is obtained by using Supplementary Equation (12).

In Ref. [11], the convention $\kappa = 2$ is used, and we thus use $x_0 = \sqrt{2}$; with this choice the survival probability reads $S(t) \sim \alpha_H/t^{1-H}$ and the value of α_H can thus be directly obtained. Now, we evaluate S in this theory for large times in Supplementary Equation (10), in this limit the function f can be evaluated for small values of y, where W admits the asymptotic behavior

$$W(y) \underset{y\to 0}{\sim} -4\ln y + A_0, \quad (14)$$

with

$$A_0 = \lim_{y\to 0}[G(y) + 4\ln y] = -2\gamma_E - 2\ln 2, \quad (15)$$

where the last equality is obtained by using Supplementary Equation (12).

Hence, the large time behavior of the survival probability reads, in this perturbative approach:

$$\begin{aligned} S(t) &\simeq \sqrt{\frac{2}{\pi}} \int_0^{\frac{1}{t^H}} dy \left(1 - \frac{y^2}{2}\right) [1 + \varepsilon(A_0 + C_0 - 4\ln y)] \\ &\simeq \sqrt{\frac{2}{\pi}} \frac{1}{t^H} \left\{1 + \varepsilon\left[A_0 + C_0 - 4\left(\ln\frac{1}{t^H} - 1\right)\right]\right\} \\ &\simeq \sqrt{\frac{2}{\pi t}} \left\{1 + \varepsilon\left[A_0 + C_0 + 4 + \ln t\right]\right\}, \end{aligned} \quad (16)$$

where we have used $t^{-H} = t^{-1/2}[1 - \varepsilon \ln t + \mathcal{O}(\varepsilon^2)]$ in the last equality. Now, expanding Supplementary Equation (4) (with $x_0 = \sqrt{2}$ and $\kappa = 2$) at order ε gives

$$S(t) \simeq \frac{\alpha_H}{\sqrt{t}}(1 + \varepsilon \ln t), \quad (17)$$

which is compatible with Supplementary Equation (16) if

$$\alpha_H = \sqrt{\frac{2}{\pi}} \left[1 + \varepsilon(A_0 + C_0 + 4) + \mathcal{O}(\varepsilon^2)\right]. \quad (18)$$

With the explicit values of A_0 and C_0 in Supplementary Equations (15) and (13), we find that this exact result is the same as the result Supplementary Equation (9) of our theory. This implies that the "decoupling approximation", in which the scaling function G is approximated by unity, is exact at order ε for $H \to 1/2$.

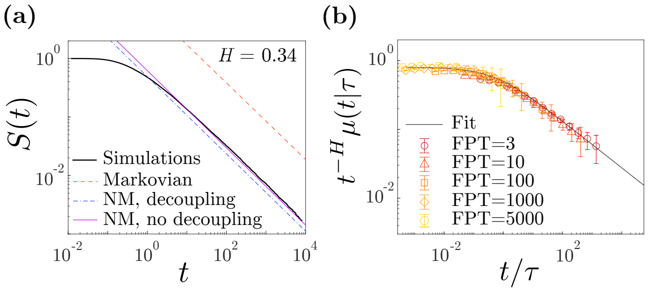

Supplementary Figure 2: (a) Survival probability for a one dimensional fractional Brownian Motion starting at $x_0 = 1$, with a target at $x = 0$, for $H = 0.34$ and $\kappa = 1$. The straight lines are the predictions of the long time behavior of $S(t)$ using the non-Markovian theory with the decoupling approximation (dashed blue line), without the decoupling approximation [red continuous line, in this case G is evaluated based on the numerical data presented in (b)], and the pseudo-Markovian approximation (red dashed line). (b) Value of $\mu(t|\tau)/t^H$ as measured in simulations, with $\mu(t|\tau) = \langle x(t + \tau)\rangle_{\mathrm{FPT}=\tau}$. We also represent the function $f(x) = 0.8/(1 + (2x)^{0.68})^{1/2}$ that reasonably fits the data.

Supplementary Note 3: More information on the scaling function G

Our exact estimate of S_0 involves the scaling function G, which is equal to unity in the decoupling approximation. Its complete analytical determination is postponed for future work. Here, we give additional information obtained by measuring it in numerical simulations in the case $H = 0.34$. Our stochastic simulations support the fact that (i) the distribution of paths in the future of a FPT is Gaussian and that (ii) the variance of trajectories in the future of the FPT is well described by its stationary value $\psi(t)$. Here, an important fact is that (i) and (ii) are valid even when conditioning by a particular (large) value of the FPT. Hence, we focus on the average $\mu(t|\tau)$,

that is the average value of $x(t+\tau)$ *given that* the FPT is exactly τ. Dimensional analysis indicates that for large t, τ, $\mu(t|\tau) \sim t^H f(t/\tau)$. This scaling behavior is confirmed on Supplementary Figure 2(b), where f displays two regimes.

Using a simple form for $f(x)$, and thus for $G = e^{-f^2/2}$, we may now evaluate S_0 from the exact equation (17) (in the main text). The result compares well with simulations in Supplementary Figure 2(a), but is only a small improvement of the simple approximate expression (18). We conclude that the expression of S_0 that is obtained within the decoupling approximation $G \simeq 1$ appears as a reliable and simple first estimate of S_0, and contains most of memory effects.

Supplementary References

[1] Meroz, Y., Sokolov, I. M. & Klafter, J. Distribution of first-passage times to specific targets on compactly explored fractal structures. *Phys. Rev. E* **83**, 020104 (2011).

[2] Weber, S., Klafter, J. & Blumen, A. Random walks on Sierpinski gaskets of different dimensions. *Phys. Rev. E* **82**, 051129 (2010).

[3] Grabner, P. J. & Woess, W. Functional iterations and periodic oscillations for simple random walk on the Sierpiński graph. *Stoch. Proc. Their Appl.* **69**, 127 (1997).

[4] Krön, B. & Teufl, E. Asymptotics of the transition probabilities of the simple random walk on self-similar graphs. *Trans. Am. Math. Soc.* **356**, 393–414 (2004).

[5] Doi, M. & Edwards, S. F. *The theory of polymer dynamics* (Oxford University Press, New York, 1988).

[6] Davies, R. B. & Harte, D. Tests for Hurst effect. *Biometrika* **74**, 95–101 (1987).

[7] Dietrich, C. & Newsam, G. N. Fast and exact simulation of stationary Gaussian processes through circulant embedding of the covariance matrix. *SIAM J. Sci. Comp.* **18**, 1088–1107 (1997).

[8] Wood, A. T. & Chan, G. Simulation of stationary Gaussian processes in $[0,1]^d$. *J. Comp. Graph. Stat.* **3**, 409–432 (1994).

[9] Dieker, T. *Simulation of fractional Brownian motion.* Master's thesis (2004).

[10] Guérin, T., Levernier, N., Bénichou, O. & Voituriez, R. Mean first-passage times of non-Markovian random walkers in confinement. *Nature* **534**, 356–359 (2016).

[11] Delorme, M. & Wiese, K. J. Maximum of a fractional Brownian motion: analytic results from perturbation theory. *Phys. Rev. Lett.* **115**, 210601 (2015).

[12] Delorme, M. & Wiese, K. J. Perturbative expansion for the maximum of fractional Brownian motion. *Phys. Rev. E* **94**, 012134 (2016).
